# Effects of Titanium Dioxide Nanoparticle Aggregate Size on Gene Expression

**DOI:** 10.3390/ijms11062383

**Published:** 2010-06-07

**Authors:** Junko Okuda-Shimazaki, Saiko Takaku, Koki Kanehira, Shunji Sonezaki, Akiyohshi Taniguchi

**Affiliations:** 1 Advanced Medical biomaterials Group, Biomaterials Center, National Institute for Materials Science (NIMS)/1-1, Namiki, Tsukuba, Ibaraki, 305-0044, Japan; 2 TOTO Ltd. Research Institute/Nakashima 2-1-1, Kokurakita, Kitakyushu, 802-8601, Japan; E-Mail: shuji.sonezaki@toto.co.jp (S.S.)

**Keywords:** nanoparticles, cytotoxicity, titanium dioxide, gene expression

## Abstract

Titanium dioxide (titania) nanoparticle aggregation is an important factor in understanding cytotoxicity. However, the effect of the aggregate size of nanoparticles on cells is unclear. We prepared two sizes of titania aggregate particles and investigated their biological activity by analyzing biomarker expression based on mRNA expression analysis. The aggregate particle sizes of small and large aggregated titania were 166 nm (PDI = 0.291) and 596 nm (PDI = 0.417), respectively. These two size groups were separated by centrifugation from the same initial nanoparticle sample. We analyzed the gene expression of biomarkers focused on stress, inflammation, and cytotoxicity. Large titania aggregates show a larger effect on cell viability and gene expression when compared with the small aggregates. This suggests that particle aggregate size is related to cellular effects.

## 1. Introduction

Nanomaterials are currently being investigated and have potential applications in various fields. They are expected to have novel physicochemical properties because of their size, chemical composition, surface structure, shape, or aggregation, and can penetrate into the body because of their small size [1]. These novel properties raise risks or safety concerns for biological systems. Some recent studies suggest that nanomaterials have potential toxicity and affect biological behavior [2–5].

Titanium dioxide (titania) is widely used, mainly for pigmentary purposes, with 70% of its production volume applied in paints, plastics, inks, foods, and toothpastes. Ultrafine-grade titania is used in cosmetics and skin care products, such as sunscreens to block ultraviolet light, as well as in catalysts. Titaniananoparticles are mostly found in aggregate form rather than alone [6]. Its aggregation, in addition to its size and shape [7], is an important factor in understanding potential cytotoxity [6]. However, the effect of the aggregate size of nanoparticles on cells remains unclear.

In this study, we prepared two sizes of titania aggregate particles, and investigated their biological activity by analyzing biomarker expression based on mRNA expression analysis. We analyzed the gene expression of biomarkers focused on stress, inflammation, and cytotoxicity.

## 2. Results and Discussion

### 2.1. Preparation of Two Different Sizes of Aggregate Titaniananoparticles

**Figure 1.**
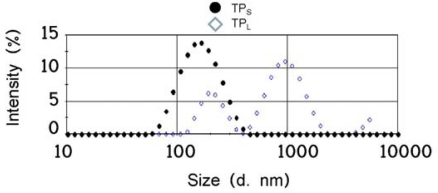
Particle size distribution of intensity measured, by dynamic light scattering analysis. Black circles show the size distribution of TP_S_, and open diamonds of TP_L_.

To determine the size effect of aggregate titania particles, two different sizes of particles were prepared from the same initial sample of titaniananoparticles. Two cell lines were exposed to these titania particles. The sizes of small and large aggregated TiO_2_ were measured to be 166 nm (PDI = 0.291) and 596 nm (PDI = 0.417), respectively (Figure 1). These particle sizes are abbreviated as TP_S_ and TP_L_, respectively.

### 2.2. Microscopic Images of Titania Particle-Exposed Cells

In this study, exposure tests were carried out for the human monocytic cell line, THP-1, and the human pulmonary endothelial cell line, NCI-H292. THP-1 cells were differentiated, before titania particle exposure, by the addition of PMA for phagocytosis.

**Figure 2.**
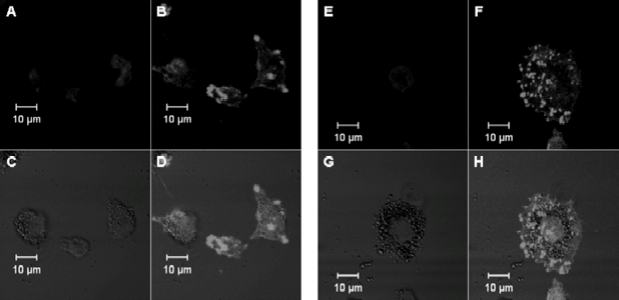
Microscopic images of TP_S_ (**A–D**) and TP_L_ (**E–H**)-exposed THP-1 cells. Titania particle-exposed cells were paraformaldehyde-fixed, and stained by Hoechst 33258 (nucleus marker; **A**, **E**) and rhodamine-phalloidin (F-actin marker, **B**, **F**). Titania particles were observed in differential interference images (**C**, **G**). (**D**, **H**) are merged images.

**Figure 3.**
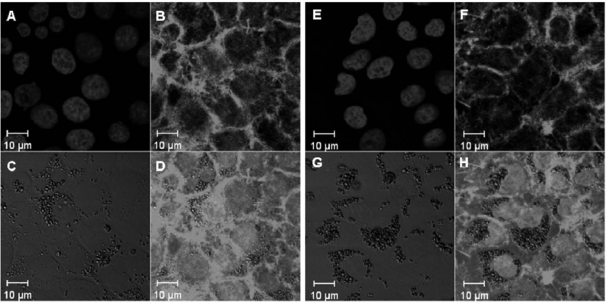
Microscopic images of TP_S_ (**A–D**) and TP_L_ (**E–H**)-exposed NCI-H292 cells. Titania particle-exposed cells were paraformaldehyde-fixed. Images represent the nucleus stained by Hoechst 33258 (**A**, **E**), F-actin stained by rhodamine-phalloidin (**B**, **F**), differential interference images to view titania particles (**C**, **G**), and merged images (**D**, **H**).

Microscopic images of titania particle-exposed cells suggested that the particles were taken up by both cell types and localized in the cytoplasmic space (Figures 2 and 3). This was the case for both TP_S_ and TP_L_ in both cell lines.

### 2.3. Cell Viability Test of Aggregate Titaniananoparticles

**Figure 4.**
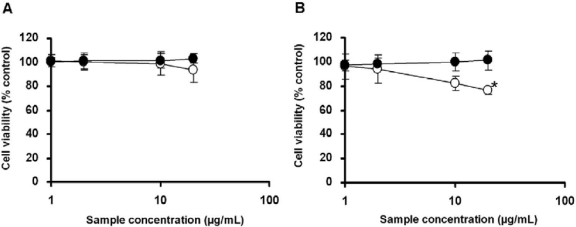
Cell viability test based on cytoplasmic ATP concentrations. THP-1 cells (**A**) or NCI-H292 cells (**B**) were exposed to each concentration of TP_S_ (closed circle) or TP_L_ (open circle). The results indicate mean ± SD, *n* ≥ 3 for each, *p < 0.01.

To analyze the cellular effect of titania particle exposure, we measured the viability of both exposed cells based on quantification of the cytoplasmic ATP concentration, which signals the presence of metabolically active cells. TP_L_-exposed THP-1 cells showed 90% cell viability, and there was a slight decrease in viability at high concentrations of TP_S_ (Figure 4A). Following 24 h of exposure to TP_S_, no apparent change in cell number was observed in NCI-H292 cells (cell viability was more than 95%), whereas cell number was decreased to around 80% following TP_L_ exposure (Figure 4B). This result indicates that TP_L_ had relatively higher cytotoxic activity compared with TP_S_.

### 2.4. mRNA Expression of Marker Genes in Titania Particle-Exposed Cells

We next investigated the mRNA expression of stress- and toxicity-associated molecular markers in titania particle-exposed cells. Selected molecular markers were heat shock protein 70B′ (HSP70B′), a universal toxicity marker; B-cell translocation gene 2 (BTG2), a DNA damage marker; cyclinG1 (CCNG1), a proliferation marker (which is related to G2/M arrest); checkpoint homolog (CHEK2), a marker related to DNA repair; chemokine (C-X-X motif) ligand 10 (CXCL10), IL6, and IL8, inflammation markers; HMOX1 and metallothionein 2A (MT2A), metabolic or oxidative markers; and tumor necrosis factor (TNF) as an apoptosis marker.

**Figure 5.**
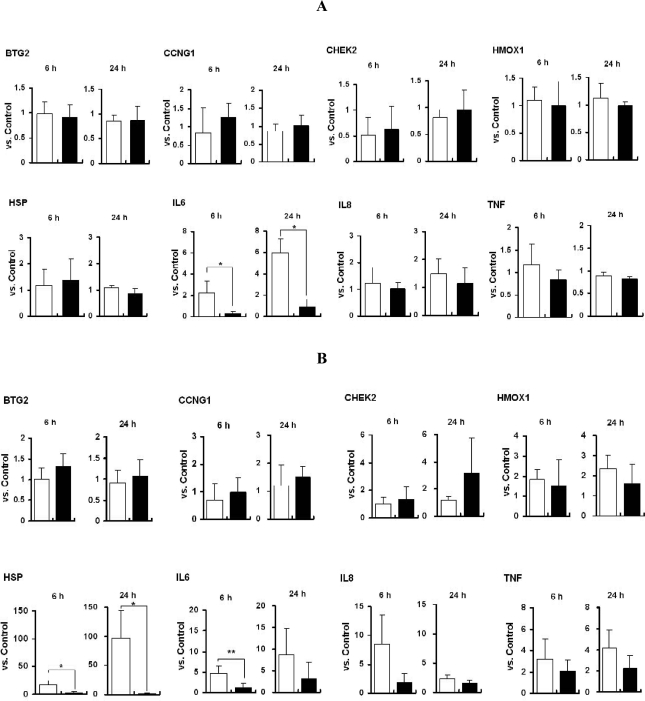
mRNA expression of stress- and toxicity- markers in TiO_2_ particle-exposed PMA-activated THP-1 cells (**A**) and NCI-H292 cells (**B**) (mean ± SD, *n* ≥ 3 for each). Cells were exposed to TP_L_ (open bar) or TP_S_ (solid bar) for 6 h or 24 h. mRNA expression was standardized by internal GAPDH (glyceraldehyde-3-phosphate dehydrogenase) expression and the relative expression level *versus* control (sterilized water added instead of TiO_2_ particles) is shown. Abbreviations: BTG2 (B-cell translocation gene 2), CCNG1 (cyclin G1), CHEK2 (CHK2 checkpoint homolog), HMOX1 (hemeoxigeanse 1), HSP (heat shock protein 70B′), IL6 (interleukin 6), IL8 (interleukin 8), TNF (tumor necrosis factor α). *p < 0.01, **p < 0.02.

In titania particle-exposed THP-1 cells (Figure 5A), IL6 mRNA was clearly induced by TP_L_ exposure. There was no apparent change in expression of other markers. In titania particle-exposed NCI-H292 cells (Figure 5B), the expression levels of IL6 and HSP depended on the size of the particles. TP_L_-exposed NCI-H292 cells showed induction of these genes, but their expression was unchanged in TP_S_-exposed cells. These results indicate that TP_L_ has a relatively greater ability to induce cellular gene expression compared with TP_s_.

### 2.5. Discussion

In this study, THP-1 and NCI-H292 cells were exposed to titania particles. The results indicate that TP_L_ affected cells more than TP_S_ did. It is necessary to clarify whether the effects of titania particles depend on the size or number of particles taken up by the cells. We thus utilized titania particles based on the weight of the particles (the particle number of TP_L_ was therefore less than that of TP_S_; the number of initial particles (Degussa P-25) was the same). According to the ATP assay, the same mass of titania particles caused a difference in cell viability. TP_L_ showed a larger effect on reducing cell viability than TP_S_ did (Figure 4). This suggests that particle aggregate size is related to the cellular effect.

Usually, the size-dependency of cytotoxity is a concern. Nano-size (<100 nm) particles produce enhanced inflammation responses when compared to larger size particles [3,8]. In this study, we compared the cytotoxic effects of small (166 nm) and large (596 nm) aggregated titania particles. Our results indicated that sub-micro large titania aggregates showed a larger effect on cell viability and gene expression when compared with the small aggregates’ effect *in vitro*. However, some researcher showed toxicity was not dependent upon particle size, but on surface characteristics [9–11]. These findings might suggest that though the size-dependency of cytotoxicity is important, the surface characteristics are more important for cytotoxic effects.

In this study, the acute biological response of titania-exposed cells was observed as changes in inflammation markers, including HSP and IL6. The heat shock protein 70 (HSP70) gene is upregulated by a wide range of cytotoxic stimulations [12–14]. Indeed, magnetic nanoparticles induce necrotic cell death, which correlates with increased HSP70 expression [15,16]. The IL6 gene is also upregulated by inflammation [17]. Taira *et al.* found, using DNA microarray technology [18], that sub-micron titanium particles induce inflammation-related genes, including the IL6 gene. Our results also suggest that HSP and IL6 are useful markers for evaluating the biological effects of nanomaterials.

It is possible that this aggregate size effect is the result of a difference in cellular uptake. The cellular uptake pathway of the particles depends on particle size and surface condition. Several cellular uptake pathways are known, including clathrin- or caveolae-mediated endocytosis, phagocytosis, pinocytosis, and macropinocytosis. Clathrin-mediated endocytosis can occur by ligand-receptor interactions or by electrostatic interactions between materials and the phosphate groups of phospholipids on the cell membrane. Clathrin forms pits with diameters of 100–200 nm, while caveolae forms pits 50–80 nm in diameter. Pinocytosis is a non-specific form of endocytosis. The diameters of vesicles formed by pinocytosis are generally less than 100 nm. Macropinocytosis [19] does not require the specific interaction of receptors. It occurs with membrane ruffling and F-actin-dependent uptake into large macropinosomes, which are 0.15–5.0 μm in diameter [20]. Upon exposure of macrophage-like cells such as THP-1, phagocytosis is expected to be the main uptake pathway. Particles ranging in size from submicron to 1 μm are appropriate for phagocytosis. In this regard, the size of TP_L_ is suitable for phagocytosis in activated THP-1 cells. We speculate that the cells incorporate too much TP_L_ nanoparticles via phagocytosis into cytoplasm, and that TP_L_ nanoparticles induce cytotoxic effects.

## 3. Experimental Section

### 3.1. Titania Particles

Titanium dioxide (TiO_2_:Degussa Aeroxide P25; 1000 mg) in 100 mL of distilled water was prepared and autoclaved at 120 °C for 20 min. The solution was cooled to room temperature, and sonicated for 10 min at 200 kHz with a strong sonicator (MidSonic 600, Kaijyo, Japan). After centrifugation (700 × *g*, 5 min) at 4 °C, the supernatant was carefully recovered and named small TiO_2_. Simultaneously, a solution without centrifugation was prepared and named large TiO_2_. The concentrations of both TiO_2_ samples were determined using a UV-VIS spectrophotometer (UV-1600, Shimadzu, Japan) by the linear relationship between TiO_2_ concentration and absorbance at 250 nm. Because small TiO_2_ was obtained at a concentration of 0.025%, large TiO_2_ was adjusted to the same concentration by the addition of distilled water, and then both particle samples were diluted by the RPMI 1640 medium supplemented with 10% fetal bovine serum. Particle size distribution was measured, by dynamic light scattering (ZetasizernanoZS, Malvern Instruments, UK), according to manufacturer’s procedures. After adjusting the concentrations, the particle sizes of small and large TiO_2_ samples were determined to be 166 nm (PDI = 0.291) and 596 nm (PDI = 0.417), respectively. After incubation at 37 °C for 24 h, the size distribution of both particle samples remained mostly unchanged. The aggregated particle sizes were reproducible and stable for at least 6 months.

### 3.2. Cell Cultures

The human acute monocytic leukemia cell line, THP-1 [21], and the human bronchial epithelial cell line, NCI-H292 [22], were cultured in RPMI 1640 medium supplemented with 10% fetal bovine serum, 100 U/mL penicillin, and 100 μg/mL streptomycin under 5% CO_2_ with 100% humidity at 37 °C. Both cell lines were cultured in the dark to avoid activation of the titania surface. For the exposure experiments, THP-1 cells were treated with 200 nM phorbol 12-myristate 13-acetate (PMA) for 48 h. To expose the cells, PMA-treated THP-1 cells or NCI-H292 cells that had been seeded 24 h prior were exposed to titania particles for 24 h.

### 3.3. Microscopic Observation

Cells exposed to titania particles were fixed with paraformaldehyde and stained by Hoechst 33258 (nucleus marker) and rhodamine-phalloidin (F-actin marker). Microscopic images of fixed cells were obtained by laser scanning microscopy.

### 3.4. Cell Viability Test

Cell viability was measured, using a CellTiter-Glo Luminescent Cell Viability Assay kit (Promega), according to the manufacturer’s instructions. For the cell viability test, 5.0 × 10^4^ THP-1 or 1.0 × 10^4^ NCI-H292 cells were seeded in each well of a 96-well cell culture plate. Titania particle suspensions were prepared at final concentrations from 0.00001 w/v% (0.1 μg/mL) to 0.001 w/v% (10 μg/mL). Each concentration of titania particle suspension was added to the cell culture medium at a 1/100 volume followed by a 24-h culture period. After 24 h of exposure to titania particles, a reagent mixture containing cell lysis solution, luciferase, and luciferase substrate, was added to the wells. The luminescence of the luciferase reaction, which depends on the cytoplasmic ATP concentration, was analyzed.

### 3.5. Gene Expression Analysis

For mRNA expression analysis, 1.4 × 10^4^/cm^2^ of THP-1 or 1.2 × 10^4^/cm^2^ of NCI-H292 cells were seeded in cell culture dishes. Titania particle suspensions were prepared at a final concentration of 0.001 w/v% (10 μg/mL). Following 6 or 24 h of exposure to titania particles, cells were detached by mechanical dissociation and utilized for gene expression analysis.

The expression levels of marker genes were determined by quantitative real-time RT-PCR as described previously [23]. Total cellular RNA was extracted from titania-exposed cells, using an RNeasy Kit (Qiagen), according to the manufacturer’s instructions. Extracted RNA was treated with DNaseI. Total cellular RNA (2 μg) was reversibly transcribed with a random hexamer primer using the SuperScript III First-Strand Synthesis System for RT-PCR (Invitrogen) according to the manufacturer’s protocol. The cDNA (2 μL) was mixed with 10 μL of 2x Master Mix from the qPCR Mastermix Plus for SYBR Green I kit (Takara) and with 10 pmol of each specific primer. The PCR procedure was as previously described [23]. Normalization was performed using the housekeeping gene, glyceraldehyde-3-phosphate dehydrogenase (GAPDH), as an endogenous control in the same reaction as the gene of interest. The primers for qPCR were as follows: for GAPDH, forward 5′-CCCCCACCACACTGAATCTC-3′ and reverse 5′-GCCCCTCCCCTCTTCAAG-3′; for B-cell translocation gene 2 (BTG2), forward 5′-AGGCACTCACAGAGCACTACAAAC-3′ and reverse 5-TGTGGTTGATGCGAATGCA-3′; for cyclin G1 (CCNG1), forward 5′-GTGGGGTGAGGTGAGCAG-3′ and reverse 5′-TGAGAGTCAGTTGTTGTCAGTACCT-3′; CHK2 checkpoint homolog (CHEK2), forward 5′-GCCAGAGAATGTTTTACTGTCATC-3′ and reverse 5′-CTTGGAGTGCCCAAAATCAG-3′; hemeoxigenase 1 (HMOX1), forward 5′-GGGTGATAGAAGAGGCCAAGA-3′ and reverse 5′-AGCTCCTGCAACTCCTCAAA-3′; Heat shock protein 70B′ (HSP70B′), forward 5′-CCGGCCCCATCATTGAG-3′ and reverse 5′-CCCATAGCATAGCCCTGACAGT-3′; Interleukin 6 (IL6), forward 5′-TGAGTACAAAAGTCCTGA-3′ and reverse 5′-TCTGTGCCTGCAGCTTCGT-3′; Interleukin 8 (IL8), forward 5′-TGCCAAGGAGTGCTAAAG-3′ and reverse 5′-CTCCACAACCCTCTGCAC-3′; Tumor necrosis factor α (TNF), forward 5′-CAGCCTCTTCTCCTTCCTGAT-3′ and reverse 5′-GCCAGAGGGCTGATTAGAGA-3′. The results from at least three independent tests were evaluated using the Dunnet multiple comparison test.

## 4. Conclusions

We prepared two sizes of titania aggregate particles and investigated their biological activity by analyzing biomarker expression based on mRNA expression analysis. Large titania aggregates showed a larger effect on cell viability and gene expression when compared with the small aggregates’ effect. This suggests that particle aggregate size is related to cellular effects.
